# Changes in the smoking status of primary care professionals and their association with rates of tobacco treatment delivery: the TiTAN Greece & Cyprus tobacco dependence treatment training programme

**DOI:** 10.1017/S1463423625100388

**Published:** 2025-08-13

**Authors:** Stavros Stafylidis, Sophia Papadakis, Paraskevi Katsaounou, Constantine Vardavas, Ioanna Tsiligianni, George Samoutis, Athina Tatsioni, Marilena Anastasaki, Charis Girvalaki, Andrew Pipe, Christos Lionis, Emmanouil Smyrnakis

**Affiliations:** 1 Laboratory of Primary Health Care, General Practice and Health Services Research, Aristotle University of Thessaloniki, Thessaloniki, Greece; 2 Clinic of Social and Family Medicine, School of Medicine, University of Crete, Heraklion, Crete, Greece; 3 Division of Prevention and Rehabilitation, University of Ottawa Heart Institute, Ottawa, ON, Canada; 4 Division of Cardiology, Faculty of Medicine, University of Ottawa, Ottawa, ON, Canada; 5 First ICU Evaggelismos Hospital Athens, National and Kapodistrian University of Athens, Athens, Greece; 6 Center for Health Services Research, School of Medicine, National and Kapodistrian University of Athens, Athens, Greece; 7 Centre for Primary Care and Population Health, St George’s, University of London Medical School at University of Nicosia, Nicosia, Cyprus; 8 Research Unit for General Medicine and Primary Health Care, Faculty of Medicine, University of Ioannina, Ioannina, Greece; 9 European Network for Smoking and Tobacco Prevention (ENSP), Brussels, Belgium

**Keywords:** Cyprus, Greece, primary care professionals, smoking, tobacco treatment, training

## Abstract

**Aim:**

This study examines the impact of a continuing medical education (CME) intervention on smoking cessation among primary-care professionals (PCPs) and explores the relationship between PCP smoking status and patient tobacco-treatment delivery.

**Background:**

High rates of tobacco use among PCPs have been reported in several European countries. PCPs who smoke are less motivated to provide cessation support to their patients.

**Methods:**

A before-after study was conducted with 228 PCPs from Greece and Cyprus. The intervention included a one-day CME training, a 2.5-hour seminar three months later, and practice tools. Expert faculty provided informal support to smoking PCPs. Changes in PCP smoking status and 5As (ask, advise, assess, assist, and arrange) tobacco treatment delivery were assessed before and six months after training. Analysis of variance (ANOVA) and analysis of covariance (ANCOVA) were used to evaluate the association between the training and PCP smoking status and 5As delivery.

**Findings:**

At baseline, 18% (*n* = 47) of PCPs were current smokers, and 39% (*n* = 66) were ex-smokers. At follow-up, 31.9% of current smokers reported quitting (*n* = 15/47; *p* < 0.001). Smoking cessation was higher among female PCPs (*p* = 0.02) and those in Cyprus and Thessaloniki (*p* < 0.01). PCPs reported increased 5As delivery at follow-up, with the highest rates among ex-smokers (>6 months) and never smokers. PCPs reported significant quitting rates following a comprehensive evidence-based training intervention. The findings suggest that addressing PCPs’ smoking status can improve both health-care provider and patient smoking outcomes.

## Background

Tobacco use remains one of the largest public health issues globally, responsible for more than 8 million deaths annually. Very high rates of smoking persist in several European Countries, with Greece reporting among the highest rates of smoking in Europe (Rachiotis *et al.*, 2017). Primary-care professionals (PCPs) can play a significant role in supporting smoking cessation among patients (Lindson *et al.*, 2021). With proper guidance, motivation, and use of evidence-based smoking-cessation treatments, patient smoking cessation can be facilitated in the primary-care setting (Vardavas *et al.*, 2009; Papadakis *et al.*, 2010). Nevertheless, many studies have documented barriers to tobacco-treatment delivery in primary care settings (Pipe, Sorensen and Reid, 2009; Saito *et al.*, 2010; Katz *et al.*, 2016).

A PCP’s personal smoking status has been identified as an important determinant of practice behaviours (Pipe, Sorensen and Reid, 2009). A meta-analysis documented a strong association between PCP’s smoking status and rates of tobacco-treatment interventions provided to patients (Duaso *et al.*, 2014). Research indicates healthcare professionals who smoke are less motivated to provide cessation support to their patients, have a less positive attitude to the value of smoking-cessation interventions, and are less likely to seek training in smoking cessation (Slater *et al.*, 2006). High rates of tobacco use are reported among health care professionals in some Southern, and Eastern European countries, as well as in Middle Eastern nations (El-Khushman *et al.*, 2008; Al-Lawati, Nooyi and Al-Lawati, 2009; Poyrazoǧlu *et al.*, 2010; Mahfouz *et al.*, 2013; Besson *et al.*, 2021). Available data indicates 38.7% of the physicians in Greece are smokers, with 83% of these physician’s reporting they started smoking before the age of 25 (Sotiropoulos *et al.*, 2007). Others have reported the smoking prevalence among nurses in Greece is 46% (Beletsioti-Stika and Scriven, 2006).

Addressing tobacco use among PCPs not only addresses the personal health status of PCPs, but may also lead to increased rates of tobacco treatment delivery among the patients within their practice resulting in a positive ‘dual effect’ (Sotiropoulos *et al.*, 2007; Duaso *et al.*, 2014; Juranić *et al.*, 2017). Furthermore, studies have documented that a reduction in smoking among physicians often contributes to a smoking decline in the general population (Vogt, Hall and Marteau, 2005; Lam *et al.*, 2011). Few interventions have targeted smoking cessation among PCPs. Interventions which seek to engage PCPs in making an attempt to quit using evidence-based treatment are needed. Some research suggests that continuing Medical Education (CME) involving pharmacological and behavioural approaches to cessation, or a combination of both, has been found to be effective in reducing the rate of tobacco use among health professionals (Beletsioti-Stika and Scriven, 2006; Movsisyan *et al.*, 2012).

The Primary Care Tobacco Treatment TrAining Network in Greece & Cyprus (TiTAN Greece & Cyprus) was a project that aimed to support the development of a network of PCPs trained in evidence-based smoking cessation treatment in Greece and Cyprus (https://titangc.uoc.gr/) the results of this project have been reported elsewhere (Girvalaki *et al.*, 2018). In a pilot study conducted among a sample of PCPs (*n* = 24), exposure to the TITAN intervention was associated with significant increases in rates of tobacco-treatment delivery (Girvalaki, Papadakis, Vardavas, Petridou, *et al.*, 2017; Girvalaki *et al.*, 2018; Papadakis *et al.*, 2022). In a pilot study, primary care professionals who smoked, expressed an interest to quit after participating in the TITAN training. At the three-month follow-up, a significant number of PCPs who reported smokers at baseline stated that they quit smoking, attributing their success to the training programme and the support received (Papadakis *et al.*, 2022). The informal nature of peer-to-peer support post-training, appeared to be a significant attribute by PCPs who smoked who might not otherwise have engaged in formal support. Given this experience with the pilot study, the TITAN Greece & Cyprus project sought to examine the effect of the TITAN intervention in the pilot study within a larger sample of PCPs.

## Aim

This paper reports on the secondary analysis of data from the TITAN Greece and Cyprus project examining the association between exposure to the CME training intervention on rates of smoking cessation among PCPs. We also examined PCP characteristics associated with smoking and successful quitting and the association between PCP smoking status and rates of tobacco-treatment delivery to patients.

## Methods

### study design

A secondary analysis of pre-post data collected as part of the TiTAN Greece and Cyprus project was conducted with follow-up occurring six months following exposure to the CME training intervention. The main results of the TITAN Greece & Cyprus project have been published separately (Papadakis *et al.*, 2022).

### Study setting

The study was conducted in four health regions in Greece (Athens, Thessaloniki, Ioannina/Kerkira, Crete) and one in Cyprus (Nicosia) between February 2017 and November 2018. In each region, a local physician coordinator (local primary care lead) identified and targeted PCPs for potential participation in the study.

### Sampling, sample size and data collection procedures

All PCPs (*n*- = 420) from each of the participating regions were contacted by email and invited to participate in the study. GPs were identified from the official listing of the local health region, A follow-up phone call was made to the professionals, to confirm their interest in participation. The eligibility criteria included: (a) PCP (GPs, nurses, health visitors) who were currently working in a public or a private primary health care setting; (b) PCP had not participated in a smoking cessation training for the last 2 years; and, (c) the primary-care practice was located in the participating geographic region. All PCPs provided written informed consent. PCPs completed a questionnaire before, immediately after and three months following the CME intervention in order to assess changes in their personal smoking status and rates of evidence-based tobacco-treatments delivery as measured by the 5As (ask, assess, advise, assist, arrange). The 5As in smoking cessation refer to a structured approach where healthcare professionals *Ask* about smoking status, *Assess* the readiness to quit, *Advise* on the benefits of quitting, *Assist* in the quit process with resources or treatment, and *Arrange* follow-up to support continued cessation (Papadakis *et al.*, 2022). To increase the response rate up to six phone calls were completed; non-respondents to the survey were offered a short telephone-based data collection during which only primary-outcomes indicators were collected.

### Training intervention

The intervention included a one-day core CME training and, three months later, a 3-hour booster seminar delivered three months later. The one-day training intervention was delivered by a faculty of national and international experts in smoking cessation, as well as a local physician champion. The training programme included the following topics: the pathophysiology of nicotine addiction, the effects of smoking on health, the role of health professionals in smoking cessation in primary healthcare settings, smoking-cessation practices, brief smoking-cessation counselling techniques, monitoring, pharmacotherapy, motivational interviewing techniques and special populations (pregnant women, adolescents, and patients with chronic diseases). The training was designed to provide 2/3 theory and 1/3 practical sessions and was based on the latest European Tobacco Treatment Guidelines (ENSP, 2016). Various training techniques were also adopted (role play, script discussion, group work, etc.) to enhance learning and facilitate practical implementation in real-world primary-care settings. Additionally, PCPs were invited to participate in 3-hour booster seminar three months following the initial CME. The purpose of the booster session was to reinforce training, offer further development of counselling skills for addressing tobacco use with patients and support the adoption of these skills in clinical practice. The training intervention was adapted from the Ottawa Model for Smoking Cessation (OMSC), which was developed and implemented at the University of Ottawa Heart Institute in Canada (Papadakis *et al.*, 2016) and was piloted in Crete (TiTAN Crete project) (Girvalaki, Papadakis, Vardavas, Pipe, *et al.*, 2017; Girvalaki *et al.*, 2018).

During the training study, investigators used informal peer-to-peer techniques to support PCPs who self-identified as smokers with personal quit support, including the offer of employing available nicotine-replacement products, as well as linking them with locally available expert stop smoking follow-up support.

### Outcome measures

**Smoking status**: The smoking status of PCPs had been assessed through survey before the training interventions. The survey asked the professionals to respond either ‘current smoker’ or ‘former smoker’ or ‘never smoker’. Six months after CME training, the smoking status was re-assessed by a telephone interview of those who reported being smokers at baseline. Professionals who reported they quit smoking were asked if the training intervention contributed to their decision to change their smoking behaviour.

**PCP performance in smoking cessation delivery**: The performance of smoking cessation delivery, as measured by 5As, was assessed through a self-reported structured survey before and six months after the training intervention.

### Statistical analysis

Descriptive analysis was used to summarize data using frequency counts and proportions (prevalence) for qualitative variables and means and standard deviation (SD) for continuous variables. The associations between smoking status and other sociodemographic variables were determined in bivariate analysis using chi-squared (x^2^) approaches to examine the trends and group differences for categorical variables and ANOVA for the continuous ones. The Mc-Nemar Bowker test was applied to calculate the significance between smoking status at baseline and after six months (Berenson, Dumas and Mazur, 2015). ANCOVA was used to determine whether the mean difference of each dimension of 5As in each subgroup (smokers, quitters, non-smokers) at baseline and after three months was statistically significant, multivariate logistic regression analysis was performed to examine the association between PCPs smoking status and their sociodemographic status (gender, region, area, practice setting and age group). A p-value <0.05 (two-tailed) was considered as statistically significant. All analyses were performed using IBM SPSS version 27.

### Ethics

The TiTAN Greece & Cyprus study received research ethics approval from the local Health authorities in Greece (1st, 2nd, 3rd, 4th, 6th, 7th Regional Health Authority-Y.P.E.), as well as from The National Bioethics Committee in Cyprus. Participation was voluntary and all PCPs who took part completed written informed consent.

## Results

### Sample characteristics

A total of 240 eligible PCPs participated in the initial 1-day CME training (recruitment rate 58.2% of eligible PCPs), and 228 provided their smoking status at baseline and were included in the present analysis. Participants’ characteristics are presented in Table 1. A response rate of 81.6% was achieved at the 6-month follow-up surveys.

**Table 1.** Demographic characteristics and smoking status of primary care providers (N = 228) at baseline overall and by smoking status

Parameter	Full sample	Current smokers	Former smokers	Non-smokers	p*
	*N* (%)	*n*(%)	*n* (%)	*n* (%)	
**Gender (** ***n*** **= 228)**					
Male	80 (35.1)	19 (22)	27 (34)	34 (44)	0.85
Female	148 (64.9)	28 (19)	57 (38.5)	63 (42.5)	
**Age group (** ***n*** **= 228)**					
< 30	3 (1.3)	1 (33.3)	0 (0)	2 (66.7)	0.62
30 – 39	51 (22.4)	12 (23.5)	17 (33.5)	22 (45)	
40 – 49	123 (53.9)	21 (17)	46 (37.5)	56 (45.5)	
50 – 59	45 (19.7)	10 (22)	18 (40)	17 (38)	
60 – 69	6 (2.6)	0 (0)	4 (66)	2 (34)	
**Geographic region (** ***n*** **= 228)**					
Crete	51 (22.0)	6 (12)	19 (37)	26 (51)	0.25
Thessaloniki	70 (30.2)	16 (23)	23 (33)	31 (44)	
Cyprus	43 (18.5)	6 (14)	17 (39.5)	20 (46.5)	
Ioannina	31 (13.4)	7 (22.5)	15 (48.5)	9 (29)	
Athens	37 (15.9)	12 (32.5)	11 (30)	14 (37.5)	
**Area of practice (** ***n*** **= 210)**					
Urban	79 (37.6)	11 (14)	29 (37)	39 (49)	**0.02**
Semi urban	65 (31.0)	20 (31)	19 (29)	26 (40)	
Rural	66 (31.4)	8 (12)	31 (47)	27 (41)	
**Practice sector (** ***n*** **= 207)**					
Private	28 (13.5)	3 (11)	11 (39)	14 (50)	0.48
Public	179 (86.5)	36 (20)	67 (37.5)	76 (42.5)	

*ANOVA test.

Statistical significance at <0.05.

At baseline, 18% (*n* = 47) of PCPs reported that they were current smokers and 25% (*n* = 66) that they were ex-smokers. From the total sample of PCPs, 64.9% (*n* = 148) were female and more than 75% (*n* = 171) were under 50 years of age. At baseline, a statistically significant relationship was found between PCPs’ area of practice (urban, semi-urban, rural) and smoking status, with PCPs in semi-urban areas reporting current smoking at significantly higher rates (p-value = 0.02, Table 1).

### Changes to PCPs’ personal smoking status

Thirty-one per cent of PCPs who reported current smoking at baseline (*n* = 47) reported they quit smoking following exposure to the training (*p* < 0.001) (Table 2). Quitting smoking following exposure to the TITAN training programme was observed among a greater proportion of female PCPs and PCPs practicing in Cyprus and Thessaloniki (p-values = 0.02 and 0.01, respectively) (Table 3).

**Table 2.** Personal smoking status of primary care providers (PCPs) at the follow-assessment

Parameter	6-month follow-up		
	Continued smoking *n* (%)	Quit smoking *n* (%)	p-value*
**PCPs smoking at baseline** **(** ***n*** **= 47)**	32 (68.1)	15 (31.9)	**<0.001**

*McNemar – Bowker Test.

Statistical significance at <0.05.

Quit smoking = Reported quitting smoking between baseline and follow-up assessment.

**Table 3.** Demographic characteristics of primary care providers reporting current smoking at baseline (n = 47), by smoking status at follow-up

Parameter	Still smoking	Quit smoking	p-value*
	*n* (%)	*n* (%)	
**Sex**			
Male	10 (55)	8 (45)	**0.02**
Female	22 (75.8)	7 (24.2)	
**Age group**			
< 30	1 (100)	0 (0)	0.52
30 – 39	7 (58.3)	5 (41.7)	
40 – 49	16 (69.6)	7 (30.4)	
50 – 59	10 (83.3)	2 (16.7)	
**Region**			
Crete	6 (85.7)	1 (14.3)	**0.01**
Thessaloniki	8 (50.0)	8 (50.0)	
Cyprus	2 (33.3)	4 (66.7)	
Ioannina	6 (100)	0 (0)	
Athens	10 (83.7)	2 (16.6)	
**Area of practice**			
Urban	9 (69.2)	4 (30.8)	0.90
Semi urban	15 (71.4)	6 (28.6)	
Rural	7 (77.8)	2 (22.2)	
**Practice sector**			
Private	2 (50)	2 (50)	0.43
Public	27 (69.2)	12 (30.8)	

*Statistical significance at <0.05.

*ANOVA test.

### Association between PCPs’ personal smoking status and rates of tobacco treatment delivery

Prior to exposure to the training intervention, rates of patient tobacco treatment delivery for three of the five 5 As (assess, assist, arrange) were significantly higher among PCPs who were ex-smokers when compared to either current smokers or never smokers (Assess 53.1% vs. 44.0% and 46.6%; *p* = 0.04, Assist 32.4% vs. 27.9% and 23.6% *p* = 0.03, Arrange 28.2% vs. 20.1% and 18.2% *p* = 0.03 respectively) (Table 4).

**Table 4.** Rates of 5As tobacco treatment delivery by PCP smoking status prior to exposure to the TITAN training

Parameter	Current smokers *n* = 47 mean %	Ex-smokers *n* = 84 mean %	Never smokers *n* = 96 mean %	p-value*
Ask	73.9	76.4	78.1	0.67
Advise	50.8	56.5	51.3	0.29
Assess	44.0	53.1	46.6	**0.04**
Assist	27.9	32.4	23.6	**0.03**
Arrange	20.1	28.2	18.2	**0.03**

*ANOVA test.

Following exposure to the TITAN training intervention, significant increases in 5As delivery were reported by PCPs regardless of their personal smoking status (Table 5). PCPs who were non-smokers (never smokers and ex-smokers) reported the highest overall rates of 5As delivery. When compared to continued smokers, non-smoking PCPs reported significant differences in treatment rates: Ask (91.6% vs 76.6% *p* = <0.001), Advise (80.9% vs 66.5% *p* = 0.001), and Assist (61.3% vs 51.3% *p* = 0.033). Both non-smokers and current smokers PCPs reported higher rates of 5As delivery at follow-up in comparison to PCPs who were recent quitters (See Table 5). While the relative rate of 5As delivery was significantly lower among recent quitters when compared to never smokers and current smokers, a large change in rates of advice (17.1% vs. 30.7% *p* = 0.155), assist (13.9% vs. 29.6% *p* = 0.109), and arrange (5.6% vs. 25.0% *p* = 0.065) was documented among PCPs who reported quitting (recent quitters) between baseline and follow-up (See Table 5).

**Table 5.** Rates of patient tobacco treatment delivery (5As) at baseline and six months after exposure to the TITAN training intervention by primary care providers’ smoking status

Parameter	Continued Smokers *n* = 32	Recent Quitters *n* = 15	Non-smoker *n* = 147	Continued smokers vs. recent quitters	Recent quitters vs. non-smokers	Continued smokers vs. non smokers
	*n* (%)	*n* (%)	*n* (%)	p-value	p-value	p-value
**Ask**						
*Baseline (T1)*	83.9	58.3	76.3	**0.001**	0.620	**<0.001**
*Follow-up (T2)*	76.6	63.9	91.6			
*Change (T2-T1)*	-7.3	5.6	15.3			
*p-value for change*	0.107	0.708	**<.001**			
***Advise***						
*Baseline (T1)*	58.1	38.9	52.7	**0.014**	0.752	**0.001**
*Follow-up (T2)*	66.5	55.6	80.9			
*Change (T2-T1)*	8.4	16.7	28.2			
*p-value for change*	0.079	0.155	**<.001**			
**Assess**						
*Baseline (T1)*	48.0	38.9	47.2	0.149	0.177	0.990
*Follow-up (T2)*	60.5	41.7	59.6			
*Change (T2-T1)*	12.5	2.8	12.4			
*p-value for change*	**0.040**	0.782	**<.001**			
**Assist**						
*Baseline (T1)*	37.4	13.9	26.7	**0.039**	0.647	**0.033**
*Follow-up (T2)*	51.3	29.6	61.3			
*Change (T2-T1)*	13.9	15.7	34.8			
*p-value for change*	**0.002**	0.109	**<.001**			
**Arrange**						
*Baseline (T1)*	29.8	5.6	21.3	0.128	0.695	0.167
*Follow-up (T2)*	48.4	25.0	58.0			
*Change (T2-T1)*	18.6	19.4	36.7			
*p-value for change*	**<.001**	0.065	**<.001**			

SD = Standard deviation.

ANCOVA p-values for differences between the adjusted means using Tukey correction.

Statistical significance at <0.05.

Continued smokers = report current smoking at baseline and the 6-month follow-up.

Recent quitters = reported quitting smoking between baseline and 6-month follow-up.

Non smoker = never smoker and ex-smokers (>6-months).

## Discussion

### Main findings

Approximately one third (31.9%) of PCPs who were current smokers at baseline reported quitting smoking six-month following exposure to the TITAN CME intervention with higher rates of quitting reported among female PCPs and geographic regions.

Additionally, we documented an increase in rates of self-reported patient tobacco treatment delivery regardless of PCP smoking status six-month following the training intervention. However, we observed different trajectories based on personal smoking status. Specifically, at follow-up higher absolute rates of tobacco-dependence treatment delivery were reported among PCPs who were never smokers or ex-smokers who quit more than six months prior were significantly higher when compared to PCPs who reported current smoking and recent quitters (<6 months). Among recent quitters (<6 months), where we saw lower overall rates of tobacco-treatment delivery, we documented a significant change from baseline for ‘Advise’, ‘Assist’ and ‘Arrange’.

The effect of the intervention on PCP smoking status may be attributed to its ability to more specifically educate primary care professionals (PCPs) on the health effects of smoking, nicotine addiction as a disease and the effective use of evidence-based tobacco dependence treatments, including behaviour change techniques and stop smoking medications – all serving to prompt and motivate cessation attempts. The informal proactive expert peer support provided alongside the training is hypothesized by study investigators as further engaging and assisting for PCPs’ in addressing their personal smoking behaviour.

Our findings are consistent with published research documenting that CME programmes substantially altered physicians smoking behaviour and also the way they counselled their patients who smoked (Lancaster and Fowler, 2000; Carson *et al.*, 2012). A study by Wu et al. examined the effects of a smoking cessation training among healthcare professionals and found a decrease in rates of smoking, from 15.9% before training to 6.3% after training; whereas the proportion of healthcare professionals, who were motivated to quit smoking increased from 41.4% at baseline to 72% after 6 months (Wu, Xing and Yang, 2010). Other researchers, evaluating an internet-based smoking cessation programme to support nurses to quit smoking, documented significant rates of smoking abstinence (45% at 6 months post registration) (Sarna *et al.*, 2009) and an increase on tobacco-treatment delivery in their clinical practice (Martín *et al.*, 2011).

Rates of quitting following exposure to the TITAN intervention were reported more frequently among female PCPs and PCPs in Cyprus and Thessaloniki; with no other significant differences were observed. The present study did not, however, assess important variables known to be associated with rates of smoking and ability to successfully stop, including the level of tobacco dependence reflected by the number of cigarettes smoked per day, and personal motivators and barriers to quitting and use of evidence-based treatment. It is hypothesized that PCPs who were not successful with quitting may have higher rates of tobacco dependence, greater barriers to quitting such as psychological stress or mental health challenges and may not have used evidence-based treatments such as pharmacotherapy. These factors are established determinants of successful quitting (Lasser *et al.*, 2000; Lindson *et al.*, 2023). Future interventions should seek to tailor treatment to PCPs personal smoking profile and potentially increase follow-up support with a focus on those PCPs who may have higher rates of dependence or barriers to cessation.

Our study adds to previously published research regarding the association between PCPs personal smoking status and rates of tobacco dependence treatment delivery to patients (Carson *et al.*, 2012). Duaso et al. found professionals who smoke were 13% less likely to offer cessation advice to their patients and 25% less likely to organize a follow-up counselling procedure for smoking cessation of patients, either in person or by phone (Duaso *et al.*, 2017). A second systematic review found that physicians who were regular smokers were less likely to advise their patients to quit smoking than non-smokers (Duaso *et al.*, 2014). A study which assessed smoking habits among Greek physicians and their impact in tobacco treatment delivery indicated that the proportion of physicians who reported counselling patients to stop smoking was lower among current smokers compared with those who never smoked or those who were former smokers (74.4% vs. 85.3% vs. 84.7%, *p* < 0.0001) (Sotiropoulos *et al.*, 2007). The TITAN project documented the highest rates of tobacco dependence delivery at baseline among ex-smokers. It is reasonable to assume that personal experience with successful smoking cessation increases the likelihood that PCPs will address smoking with patients. Interestingly, while we saw an increase in rates of self-reported tobacco treatment among recent quitters (<6 months), the relative rates of delivery were much lower than ex-smokers and never smokers. This may suggest that PCPs who recently stop smoking do not necessarily intervene at high rates until they have experienced an extended period of successful cessation. More research is required to confirm and validate the underlying factors that may explain these trends.

### Strengths and limitations

The present study has strengths and limitations. We involved a sample of PCPs which is, to our knowledge, the largest sample completed within Greece and Cyprus. Participants were selected from different regions of Greece and Cyprus and high rates of follow-up are documented. Our study also has some limitations. This study relies on secondary analysis, which, while informative, may have limitations in fully identifying causal relationships due to its explanatory nature. Moreover, it is possible that recruitment and reporting bias may have occurred. Recruitment bias is related to the purposive sampling and voluntary nature of participation in the study, as well as that the group of participants may consisted of those with a special interest in the subject. In addition, the participants who reported current smoking at baseline may have had other factors contributing to their personal motivation to quit smoking, which were not assessed in this study (Stafylidis *et al.*, 2024). Reporting bias is related to the self-reported nature of the data collected. The outcomes for personal smoking status and 5As are self-reported by PCPs and the possible under-reporting rates of smoking behaviour could not be determined. The limitations mentioned above may result in an overestimation of the impact of the intervention on PCPs’ smoking status and practice behaviour. Future research should consider validated methods for documenting smoking status such as CO2 measurement and patient-level measurement of 5As delivery. While our sample size was large the proportion of current smokers at baseline was 18% and as such limits the opportunity for within and between group comparisons. Consequently, future studies should consider a comparative approach to further validate these results.

### Implications for clinical practice, education or further research

This analysis provides critical insights into how the smoking behaviours of primary care professionals (PCPs) can significantly influence the delivery of tobacco treatment services. By highlighting the correlation between PCPs’ smoking status and their ability to effectively support patients in cessation efforts, this study underscores the necessity for targeted training programmes that not only enhance the skills of healthcare providers but also promote their personal cessation efforts, thereby improving overall treatment outcomes in diverse healthcare settings (Dannapfel *et al.*, 2023; Stafylidis *et al.*, 2024). This research is also relevant for generalization, as it emphasizes the importance of integrating personal health behaviours into professional practice, which can inform global strategies for tobacco control and treatment delivery (Leal *et al.*, 2022; Britton *et al.*, 2023). Particularly, the study documented that 18% of PCPs were current tobacco users. Our results show that educational interventions supporting evidence-based tobacco-treatment interventions may positively impact PCPs’ personal smoking status, and underscore the pivotal role of PCPs as influential figures in encouraging and supporting patients’ smoking-cessation efforts (Wu, Xing and Yang, 2010; Papadakis *et al.*, 2020). The demonstrated impact of CME interventions on PCPs’ smoking cessation highlights the potential for targeted educational programmes to contribute not only to enhanced knowledge and skills but also to personal behavioural change. The observed increase in 5A delivery rates across all PCP subgroups further emphasizes the tangible benefits of such interventions. Personal health status, beliefs about quit-smoking supports, and low self-efficacy appear to influence patients’ motivation to make an aided quit attempt (Manoharan *et al.*, 2022). These findings suggest that integrating smoking cessation training into the professional development of PCPs can have a dual effect, positively influencing patient care and fostering personal behaviour change. As we move forward, future research should delve into understanding the barriers and facilitators that shape PCPs’ engagement in smoking-cessation practices within their daily clinical routines, thereby informing tailored strategies for more effective implementation and sustained impact.

## Conclusions

PCPs reported quitting at significant rates following their exposure to an evidence-based training intervention. The study findings support the dual role of PCPs as both influencers of patient behaviour and being beneficiaries of behavioural change themselves. Our results emphasize the importance of targeted smoking-cessation educational strategies, such as the TITAN intervention. The study also adds to the current body of evidence from primary care in Greece and Cyprus and its findings could be instrumental for the primary care reforms under debate in both countries.
